# Numerical and Experimental Modal Analysis of a Gyroid Inconel 718 Structure for Stiffness Specification in the Design of Load-Bearing Components

**DOI:** 10.3390/ma17143595

**Published:** 2024-07-21

**Authors:** Katarina Monkova, Sanjin Braut, Peter Pavol Monka, Ante Skoblar, Martin Pollák

**Affiliations:** 1 Faculty of Manufacturing Technologies with a seat in Presov, Technical University in Kosice, 080 01 Presov, Slovakia; peter.pavol.monka@tuke.sk (P.P.M.); martin.pollak@tuke.sk (M.P.); 2 Faculty of Technology, Tomas Bata University in Zlin, Nam. T.G. Masaryka 275, 760 01 Zlin, Czech Republic; 3 Faculty of Engineering, University of Rijeka, Vukovarska 58, 51000 Rijeka, Croatia; ante.skoblar@riteh.uniri.hr

**Keywords:** modal analysis, natural frequencies, gyroid, Inconel 718, stiffness

## Abstract

The study aims to investigate the modal properties of a 60 × 70 × 80 mm gyroid structure made of Inconel 718 with 67.5% porosity. The geometry model for sample production was created using the software PTC Creo, whereas the geometry model for numerical analysis was created using the Python application ScaffoldStructures. FE analysis was performed using ANSYS 2024 R1 software. Free boundary conditions were used in experimental modal analysis to ensure feasibility. The analysis identified the first four natural frequencies ranging from 10 to 16 kHz. The results revealed that the first natural frequency corresponds to the first torsional frequency about the *Z* axis, the second to the first flexural mode in the XZ plane, the third to the first bending mode in the YZ plane, and the fourth to the first torsional mode about the *X* axis. Small differences between the results of numerical and experimental modal analysis can be attributed to geometric errors in the manufactured sample, careless removal from the platform, and due to reduction in the complexity of the numerical FE model. Employing modal analysis of a component, the stiffness of a lightweight component can be revealed. In the case of the sample with the cellular structure of gyroid type, relatively high stiffness regarding the material savings was identified, which can be advantageously used in many applications.

## 1. Introduction

Today’s low-mass, structurally more complex, and compact products are the result of the application of computer-aided development with algorithmized sophisticated methods of analysis, synthesis, and optimization of mechanisms as well as the result of the use of high-strength materials obtained via new production technologies. Thanks to powerful drives, products, and devices working at high operating speeds—where inertial properties, uneven distribution of rotating masses, gear nonlinearities, and the effects of external dynamic excitation on frequency responses are significantly manifested due to low material damping—products are prone to acquiring a resonant state [1].

New generation products are expected to withstand dynamic loads at the limit of the elastic properties of materials, maintain stable dynamic properties resistant to random changes in external influences, and withstand gradual changes in internal properties, all in response to increasingly demanding requirements to reduce costs and shorten development time. They will exhibit consistently high performance, a longer lifespan, and autonomously functioning control systems, ensuring their reliable operation in challenging conditions with minimal maintenance requirements, all while maintaining a low price. In those cases in which oscillation is undesirable, the products should be increasingly quiet and dynamically balanced, with minimal excitation forces in the elastic-damping elements of the storage. On the other hand, if oscillation is necessary, we require that the working members consume as little as possible to generate oscillation energy [2,3,4,5,6].

Modal analysis therefore plays a significant role in predicting the behavior of newly developed machines and devices with the implementation of new types of porous materials in their supporting and structural parts, while these porous materials can bring extraordinary properties to the system characterized by low noise, excellent damping, light but rigid skeleton, and the expected mechanical properties.

## 2. State of the Art

Modern technologies based on an additive approach have made it possible to produce lightweight components with regularly distributed cellular structures whose properties can be predicted. At the same time, the development of computer technology helped support the solution of calculation models related to bodies with complex configurations, including bodies with implemented TPMS (triply periodic minimal surfaces) structures. The mechanical properties of the components built on the base of porous structures depend primarily on the material from which they are produced, but many other aspects related to the topology and manufacturing conditions need to be taken into account when their characteristics are assessed [7].

For the components that operate under specific extreme conditions—such as, e.g., in aggressive chemical conditions—the material has to be selected very carefully. Such a situation occurs in manufacturing machines operating at high temperatures within incinerators or industrial enterprises/workshops for producing and processing metals (smelters, rolling mills, presses, hammers, etc.), for which the criteria for mechanical properties and material selection are very demanding. One of the most suitable choices is Inconel 718 [8,9,10].

Many researchers have tried to analyze the mechanical properties of porous structures made from Inconel 718.

Wang et al. [11] dealt with the mechanical properties under compressive loading of tetrahedral and diamond structures made from Inconel 718 alloy using selective laser melting (SLM) technology. It can be stated based on their achievements that both types of structures showed all three phases of behavior typical for ductile porous materials, namely a linear elastic deformation stage, a plastic plateau stage, and a densification stage. Unlike the tetrahedral structure, which revealed elastic and plastic deformation stages, the diamond porous structure had three stages: elastic deformation, plastic deformation, and densification. At the same time, it can be said that for the diamond structure, better steadiness in the stress distribution was typical under compression compared to the tetrahedral structure. Similarities can be found in their behavior under loading, which shows good plasticity and toughness, while fundamental dissimilarities between them were observed in the plateau region in its height and length [11].

Zhao, Z. investigated the effect of heat treatment on the microstructure and mechanical properties of 54% porosity samples, which were fabricated similarly to Wang’s research [11] from Inconel 718 using selective laser melting (SLM) [12]. Austenite with cellular and columnar crystal morphology was formed in the IN718 alloy. The studied porous structure showed a yield strength of 237 MPa at a strain of 3% before heat treatment, while the maximum deformation was 28%. After heat treatment, the properties improved slightly, the yield strength by about 12 MPa and Young’s modulus by 6.36 GPa, which was probably due to the uniform distribution of the γ″ and γ′ phases [12].

The researchers under the supervision of Wang X [13] scrutinized distinct lattice structures using Inconel 718 superalloy. The honeycomb porous lattice displayed impressive tensile strength, maintaining its robustness at relative densities above 60% with a mere 3.29% deviation from the original specimen. In contrast, the I-WP lattice showed a direct relationship between elongation and relative density. The study also provided valuable insights into deformation behaviors within porous structures, revealing the distinct behaviors of stretching, bending, or combined deformations under various conditions. These specific findings inform the design of lightweight, heat-resistant, load-bearing structures [13].

In the study by Kladovasilakis [14], the research is focused on orthopedic hip implants with functionally graded lattice structures made of Inconel 718 superalloy that was investigated through finite element analysis. The study explored the mechanical performance of these structures and their suitability for orthopedic implants. Notably, the Schwarz Diamond lattice structures exhibited the best mechanical behavior in both simple and functionally graded implants, with factors of safety of 1.01, 1.79, and 2.08 for Voronoi, gyroid, and Schwarz Diamond, respectively. Topology optimization led to a 38% reduction in implant weight, and the intramedullary stem featured 50% porosity to support tissue regeneration.

The research of Yang et al. [15] used SLM technology to create Inconel 718 CLL materials with good repeatability. Slight deviations in struts/pillars were observed due to powder adhesion. Inconel 718 exhibited a high yield strength of 796.46 MPa and a fracture strain of 0.22 greater than 3D-printed aluminum/iron/titanium alloy. Compression results showed distinct behaviors: graded CLL outperformed graded BCC by about 50% in specific energy absorption. Vertical pillars in CLL designs enhanced compressive properties, while BCC designs displayed a more stable deformation mode. Graded CLL achieved approximately 1.2-times-higher specific energy absorption than graded BCC. These findings highlight the potential of functionally graded CLL materials for industrial applications [15].

Monkova with her team investigated the mechanical properties of mono- and double-gyroid structures made from Inconel 718 using DMLS technology. They examined how crosshead speed and varying volume ratios affect compression properties in these structures. Notable findings include the highest Young’s modulus of 58 GPa in sample G10_1 and the lowest of 33 GPa in G20_3. Specimen G10_1 also exhibited the best compression properties with a yield strength of 874 MPa and a stress of 1089 MPa at the first peak. Crosshead speed didn’t significantly influence the structures, while energy absorption increased with volume ratio. Interestingly, double-gyroid structures at the same volume ratio performed less effectively, likely due to thinner walls and intersections that created weaker areas [16].

In another study, Monkova et al. conducted an extensive study on 3D-printed Inconel 718 specimens, focusing on tensile properties. Dog-bone samples were printed in X and Y orientations. Results indicated similar tensile properties, with an average yield strength (Rp_0.2_) of approximately 1338 MPa, tensile strength (Rm) of around 1516 MPa, and an average elongation (A35) of 14%. Fractographic analysis revealed a consistent failure mechanism marked by fine-sized dimples, trans-granular facets, and cavities. These findings offer valuable insights into additive manufacturing [17].

The researchers compared the performance of different lattice structures made by laser powder bed (L-PBF) from Inconel 718. The properties were evaluated using modal analysis. They found that triple periodic minimum surface (TPMS) lattice structures have a better strength-to-volume ratio than rod lattices. Lattice structures have several advantages over traditional structures. They are lighter, which can lead to weight and fuel savings. They also have a greater strength-to-weight ratio, meaning they can withstand greater loads for the same weight [18].

The researchers in the team of Beghini [19] proposed a model that helps designers design mechanical components with desired vibration-damping properties. The model uses an elastic metamaterial to simulate lattice structures (LS). This allows designers to easily adjust the parameters of the LS core to control and optimize the global modal frequencies of the entire geometry. The researchers validated their model through experimental testing and concluded that it is suitable for implementation into an automated tool for designers [19].

Several other studies have addressed the mechanical properties of cellular structures [20,21,22,23,24,25,26] but very few of them have investigated the dynamic characteristics of complex cellular structures made of Inconel 718, which can bring to machines operating under specific high-temperature conditions and high dynamic loads more efficient properties, such as better stiffness-to-weight—the machines can have a lower weight while maintaining the required stability, and at the same time, the machines can feature excellent damping along with outstanding energy and sound absorption properties. Calculation of the dynamic characteristics of the basic nodes and the entire flexible system of the production machine when designing a new or modernizing an existing machine allows one to choose the best design solution and dynamic parameters of the machine from several variants.

This is why the authors decided to deal with modal properties of the gyroid structure that belongs to so-called triply periodic minimal surfaces (TPMS) made of Inconel 718. Research of the modal parameters of a sample with a gyroid structure, specified numerically and verified by experimental analysis, will allow in the future (when implementing the gyroid structure into the supporting parts of the production equipment) to set the correct boundary conditions and to use numerical analyses to expertly predict the properties of the equipment even before its production itself, which they eliminate attempts to make production equipment in the wrong way. A right-designed virtual model with specified properties will also be able to serve as a virtual twin for monitoring changes in its behavior, predicting the failure of worn parts (or other damages), and also for appropriately selected maintenance time. Based on the specification of the natural frequencies, it is possible to calculate the stiffness of the load-bearing components and at the same time it is possible to prevent the system from resonating.

## 3. Methods and Materials

### 3.1. Materials

One subgroup of materials suitable for the design of load-bearing components of production machines are structures formed by steel or carbon covers and an aluminum foam core. The structure makes it possible to achieve a certain flexibility while maintaining a low weight. In the form of sandwich materials, it is possible to create semi-finished plates that can be joined into more complex structures of production machine parts. This complexity of shapes and joining of plates to each other could be now (or soon) directly replaced by components with a lightweight core made of cavities (cells) with a controlled arrangement, which is manufactured using an additive approach [27].

If the machine works in operations with extreme temperatures, it is possible to use lightened Inconel 718. Therefore the authors decided to prepare TPMS samples with a gyroid structure from this material and use modal analysis to determine its stiffness.

The gyroid surface is characterized by a geometry that can be described by Equation (1) [28]

(1) $$\sin\left(x\right)*\cos\left(y\right)+\sin\left(y\right)*\cos\left(z\right)+\sin\left(z\right)*\cos\left(x\right)=0$$

Inconel can be used in environments from cryogenic up to 700 °C. Throughout this entire range, it exhibits exceptionally high yield, tensile, and creep–rupture properties. It also shows excellent tensile and impact strength. The alloy is made up of 50–55% nickel + cobalt (with cobalt limited to 1% max) and 17–21% chromium. This combination gives the material its corrosion-resistance properties. This includes good oxidation resistance, enabling it to withstand corrosive media present in many of its applications. The remainder of the composition includes niobium + tantalum (4.75–5.5%), molybdenum (2.8–3.3%), and titanium (0.65–1.15%), plus other balancing elements. One of the outstanding features of Inconel 718 alloy is that it is extremely versatile and easy to work with. It demonstrates excellent welding characteristics, particularly concerning its resistance to post-weld cracking [29,30,31].

**Figure 1:**
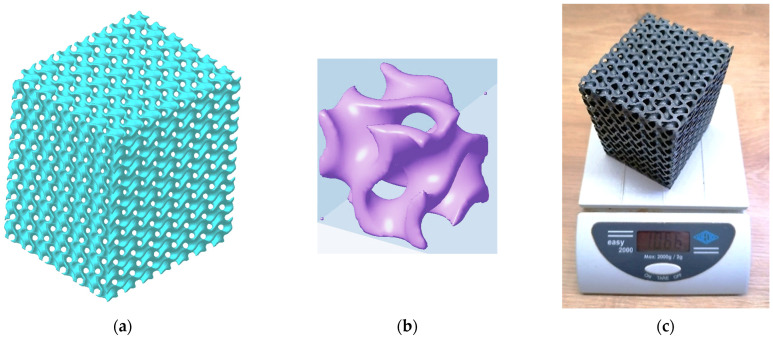
Gyroid sample; (**a**) a virtual 3D model 60 × 70 × 80 mm; (**b**) a basic cell 10 × 10 × 10 mm; (**c**) an experimental sample made of Inconel 718.

The sample for analysis was made as a structure of the gyroid type sized 60 × 70 × 80 mm (Figure 1a) with 6 × 7 × 8 cells that correspond to the basic cell dimensions 10 × 10 × 10 mm (Figure 1b) and 67.5%. The porosity of the sample was computed according to Equation (2) [16]

(2) $$Vp=1-\frac{material\;volume}{total\;sample\;volume}\;$$

Software PTC Creo 8 was used for the virtual 3D model preparation, while the porosity (material volume) was driven with a wall thickness. Exported data in *.stl format were used for the sample production.

DMLS machine EOS EOSINT M290 was employed for the sample production, while layer thickness was 0.04 mm which corresponds to the machine setup recommended by a producer for the given material Inconel 718. Before removal from the build platform, the samples were heat-treated according to the procedure according to AMS 5664 [32]—annealing at 1065 °C for 1 h and cooling with inert gas followed by tempering at 760 °C for 10 h, cooling in a furnace at 650 °C for 2 h, and holding at 650 °C for 8 h. Subsequently, the samples were cooled in an environment with inert gas to ambient temperature [33]. A produced sample with a weight of 1.066 kg is presented in Figure 1.

### 3.2. Stiffness of a Machine

The design of production machines is made up of a system of elements and nodes that are mutually distributed and connected in different ways, which are determined by the fulfillment of the basic mission of the production machine—the implementation of the relevant technological process. Technological rigidity is decisive for the accuracy of the machine’s work. Therefore, when designing the machine, it is necessary to deal with the question of the overall rigidity of its structure [34,35].

**Figure 2:**
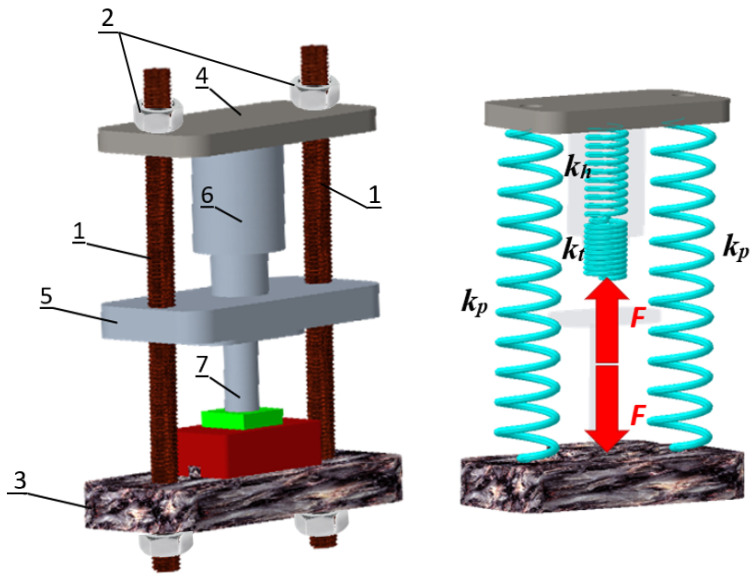
The principle of the total stiffness calculation of a production machine, where 1—pillars, 2—clampers, 3—base, 4—crossbar, 5—ram of the high-energy-rate forging hammer (slide), 6—hydro-motor, and 7—tool.

The total rigidity of the machine structure in a precisely defined location and direction can be calculated as the resulting spring constant of one spare spring, with which we can replace the machine in question. In general, this resulting spring can be created as a replacement for series, parallel, or combined springs replacing the respective partial stiffnesses of the bodies. The calculation of the total stiffness of the production machine can be presented on a simplified construction of a hydraulic power forging machine shown in Figure 2, where 1—pillars, 2—clampers, 3—base, 4—crossbar, 5—ram of the high-energy-rate forging hammer (slide), 6—hydro-motor and 7—tool. To simplify, the foundation, cross member, and ram will be considered as perfectly rigid bodies. If the pillars are replaced by springs with stiffness *k_p_*, the hydraulic motor by a spring with stiffness *k_h_* and the tool by a spring whose stiffness is *k_t_*, then the machine can be visualized using the spring model as follows [35]:

The resulting stiffness of the investigated hydraulic high-energy-rate forging hammer will be

(3) $$k=\frac{2k_{p}k_{h}k_{t}}{k_{h}k_{t}+2k_{p}(k_{h}+k_{n})}$$

Stiffness as a function of the load can be considered linear because, in the case of the loads to which production machines are subjected in operation, Hooke’s law applies [35].

Although explicit mathematical models can be established and solved in a closed form only for dynamic systems with one degree of freedom of movement, the acquired knowledge is the key to understanding the more complex dynamic properties of systems with several degrees of freedom of movement. Therefore, modal analysis also plays a significant role in the prediction of the behavior of newly developed machines and equipment with new types of porous materials that can bring the system extraordinary properties characterized by lower noise, excellent damping, and light construction with the expected mechanical properties [36].

### 3.3. Modal Analysis

Currently, modal analysis is considered a modern branch of dynamics, which is of great importance in technical diagnostics. The goal of modal analysis in structural mechanics is to determine the natural mode shapes and frequencies of an object or structure during free vibration. The implementation of modal analysis in practice dates back to the end of the 1980s. It was during this period that hardware and computer programs operating on the principle of the finite element method flourished. Thanks to the possibility of determining the resulting modal properties of the investigated system, it is possible to obtain a complete dynamic description of the mechanical system [37].

When solving tasks of modal analysis, it is possible to use different approaches built on the foundations of dynamics. To obtain the natural frequency, eigenvalue analysis can be performed, which is a mathematical operation that solves the dynamic properties of a system using its characteristic equation, composed of the system’s stiffness and mass distribution. Another approach is the solution of Lagrange’s second-order equations of motion, where no external forces act on the dynamic system. The general form of Lagrange’s second-order equations of motion is [38]

(4) $$\frac{d}{dt}\left(\frac{\partial E_{K}}{\partial \dot{q}}\right)-\frac{\partial E_{K}}{\partial q}+\frac{\partial E_{D}}{\partial \dot{q}}+\frac{\partial E_{P}}{\partial q}=0$$

where *q* is a generalized coordinate of the path (for a translational motion in the *x*-axis direction *q* is represented by *x*, for rotational/torsional motion, it is *φ*); $\dot{q}$ is a generalized coordinate of speed; and *E_K_*, *E_D_*, and *E_P_* are the kinetic, dissipative, and potential energy respectively.

**Figure 3:**
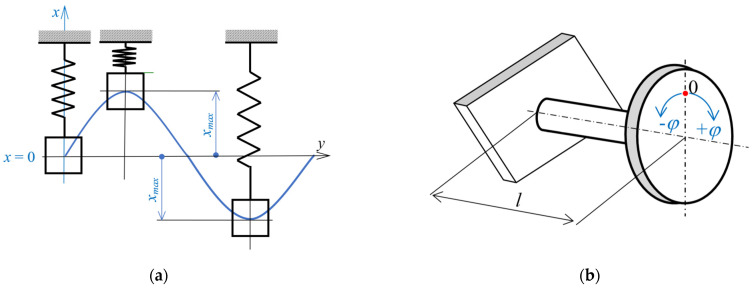
A mathematical model; (**a**) translational motion; (**b**) torsional motion.

For an undamped system with one degree of freedom of movement, the model shown in Figure 3 can be established.

The equation of motion can be written in the generalized way as:

(5) $$\ddot{q}+\Omega_{n}^{2}q=0,$$

where a natural frequency $\Omega_{n}$ can be expressed by Equation (6):

(6) $$\Omega_{n}=\sqrt{\frac{k}{m}},$$

or for torsion by Equation (7):

(7) $$\Omega_{n}=\sqrt{\frac{k}{J}},$$

where *k* is the positive stiffness constant of the system expressed in units such as N/m; *m* is mass (kg) (Figure 3a), and *J* is the mass moment of inertia to the axis of rotation (Figure 3b).

In the case of a component loading by torsion (Figure 3b) when the moment *M* is acting, a corresponding deformation of the component is given by the angle of torsion [35]

(8) $$\varphi =\frac{Ml}{GJ}$$

where *l* is the twisted length of the component and *G* is the shear modulus of elasticity of the material in shear. The stiffness of the specific component is expressed in such cases by Equation (9)

(9) $$k_{T}=\frac{M}{\varphi \;}=\frac{GJ}{l\;}$$

from which it is clear that the torsional stiffness is directly affected by the material used and the cross-sectional shape of the twisted component, while the twisted length affects the stiffness inverse proportionally.

In the case of free torsional vibrations of a beam of square cross section, free at both ends, the first natural frequency is equal to [39]

(10) $$\omega_{1}=\frac{\pi }{l\;}\;\sqrt{\frac{Gl}{\rho I_{p}}}$$

where *I*_p_ is the polar moment of inertia of the cross-section and *ρ* the specific density of the material. The denominator *ρI*_p_ is equal to the *J*_p_ which is the mass moment of the beam’s cross section in regards to the longitudinal beam axis for homogeneous distribution of the mass in the structure volume.

Based on known geometric parameters, the torsional coefficient *I* can be calculated, and based on the measured first normal frequency, the torsional stiffness according to Expression (10) can be defined.

Although practical systems have multiple degrees of freedom (MDOF) and have some degree of nonlinearity, they can generally be represented as a superposition of a single degree of freedom (SDOF) linear model and will be developed in this manner.

#### 3.3.1. Numerical Analysis

The dynamic stiffness and stability of a mechanical system, i.e., a production machine, can be defined as its resistance to oscillation, which is the response system to external time-varying stimuli. Determining the stability of complex systems with continuously distributed weight is often very imprecise due to the simplification of mathematical calculation models. Therefore, the finite element method (FEM) is currently used. Its advantage is primarily the fact that it enables the calculation of a dynamic system with continuously distributed mass for solving a system with a finite (albeit relatively large) number of degrees of freedom, basically using the mathematical apparatus to solve the generalized problem of eigenvalues for the mass and stiffness matrices of the system with computer aid [40,41,42].

**Figure 4:**
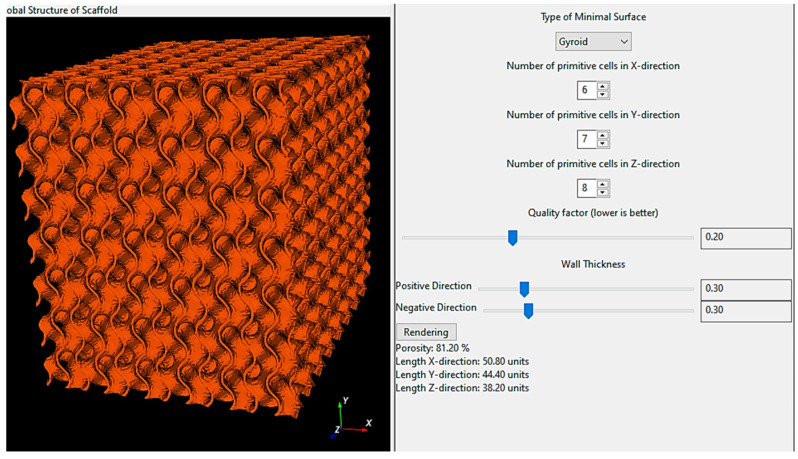
The user interface of the Python application ScaffoldStructures.

To generate a model for the finite elements analysis (FEA), the usage of the Python application ScaffoldStructures proved to be the most effective (Figure 4) of all the methods considered for generating the geometry.

The porosity of the model is adjusted with the “wall thickness” slider. The optimal value of the quality factor is 0.2 for gyroids. A smaller value of the quality factor means a more precise model but with more facets, which ultimately results in a finer mesh of finite elements. ScaffoldStructures prepare geometry in relative units. First, the dimensions of the original model should be set to then scale the model according to the desired final dimension (60 × 70 × 80 mm). After material definition (Inconel 718) a total mass of geometry can be checked.

When the model was imported to Ansys 2024 R1 software, the facets were checked and if necessary repaired with the AutoFix command.

The model was meshed with tetrahedron finite elements and Patch independent algorithm with 10,245,313 elements and 2,276,238 nodes. Within the FEA, the values listed in Table 1 for the material properties and ambient conditions were used.

#### 3.3.2. Experimental Analysis

**Figure 5:**
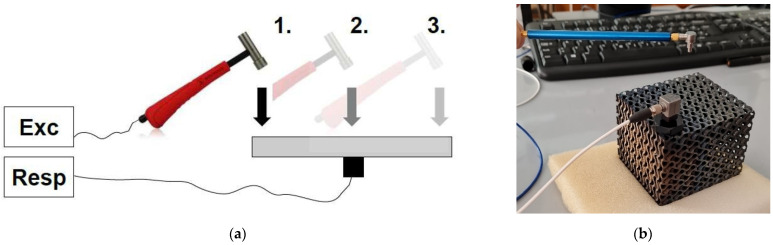
Experimental modal analysis; (**a**) principle of SISO test; (**b**) configuration of the gyroid sample testing.

To verify data obtained by the finite elements method, the experimental modal analysis (EMA) was performed that was done using the DEWESoft SIRIUS DAQ system, which offers a 2 × 24 bit AD resolution and 200 kHz sampling rate. Data Acquisition System (DAQ) with DEWESoftX 2020.1 software were used for the measurement and analysis of acquired signals. The impact hammer PCB Piezotronics, model 086E80, was employed for the excitation of the sample, and the PCB model 352C34 accelerator was used as the sensor. To perform modal characterization of the mechanical structure i.e., to determine natural frequencies and corresponding damping ratios, the frequency response function (FRF) at frequencies from 1 Hz to 15 kHz was selected as a basic tool. The sample was placed onto the soft foam and considering mounting the accelerometer by gluing, single-input–single-output testing (SISO) was selected (Figure 5a) [43,44,45]. In this operation mode, there is one acceleration sensor mounted in a fixed position on the structure and the modal hammer is moving through the excitation points. The experiment configuration is shown in Figure 5b.

## 4. Results and Discussion

**Figure 6:**
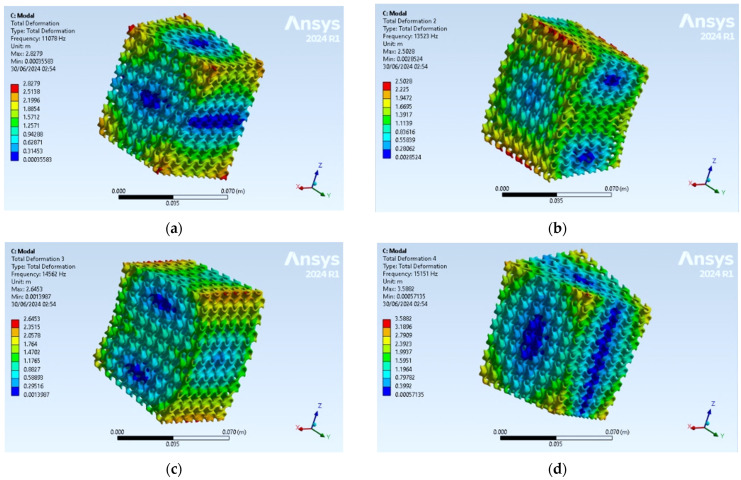
Natural shapes correspond to the first four natural frequencies, (**a**) 1st natural frequency, 11,078 Hz; (**b**) 2nd natural frequency, 13,523 Hz; (**c**) 3rd natural frequency, 14,562 Hz; (**d**) 4th natural frequency, 15,151 Hz.

The first natural frequencies obtained with the numerical approach in the ANSYS 2024 R1 software are displayed in Figure 6. The first natural frequency is the first torsional frequency about the *Z* axis (for the weakest), the second numerical natural frequency corresponds to the first flexural mode for the weakest cross section (and weakest second moment of area—moment of inertia *Ix*), the third numerical natural frequency corresponds to the first bending mode in YZ plane, and the fourth natural frequency corresponds to the first torsional mode about the *X* axis.

**Figure 7:**
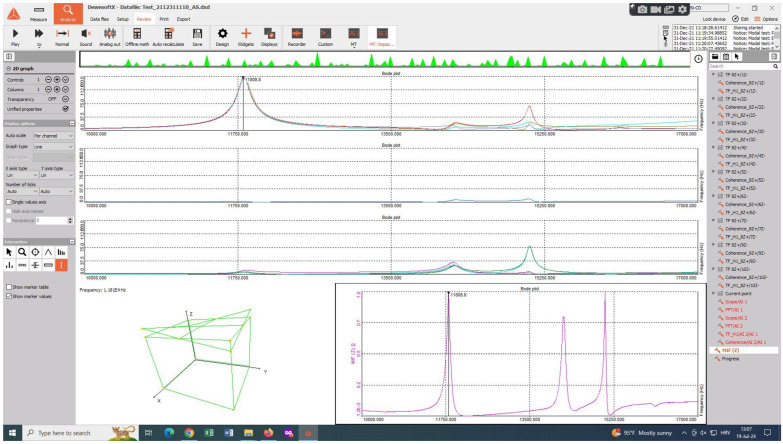
Modal testing with DeweSoft X software.

As can be seen in Figure 7, during the experimental analysis, all test hammer hit points were on the same plane as the measuring point on which the accelerometer was placed. A 9-point array was chosen for hammer hits, and the 10th point was designated as the accelerometer measurement location. Figure 7 shows the results in the form of Bode plots. The top three plots refer to Bode’s responses to hammer strikes at three points each, with each point struck three times. The bode plot in the lower right corner represents the average contribution of all 9 impact points. In the lower-left corner, a 3D animation of the vibration form for the selected natural frequency is displayed.

A comparison of natural frequencies obtained numerically and experimentally is shown in Table 2.

**Figure 8:**
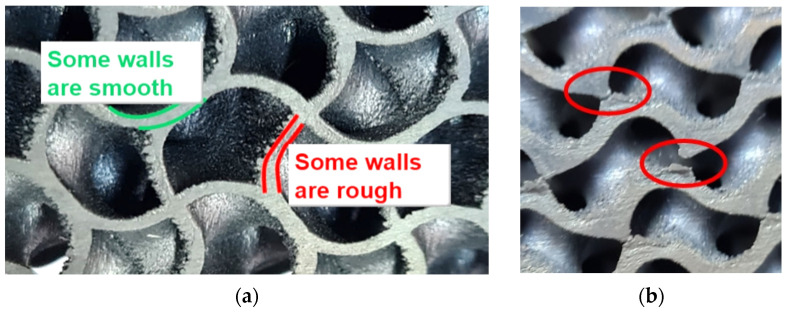
Irregularities in printed geometry of produced samples, (**a**) errors in the geometry; (**b**) interruption of the structure geometry.

The highest relative error in the range of measured natural frequencies is 6.53% at the first natural frequency, which can be considered acceptable for the given conditions. The differences that arose during the experimental modal analysis were caused by errors in the geometry of the realistic production effects since, e.g., some walls were smooth and some were rough (Figure 8a). On the rough walls, particles contribute to mass (inertia) properties but not to stiffness property, while thinned walls cause a decrease in effective stiffness. The variance was very probably caused also inappropriate removal from the platform when a wall was destroyed, and the geometry was not continuous (Figure 8b). Smaller deviations in the results can also be expected due to the sensor gluing procedure as well as the fact that the ideal free boundary conditions considered in numerical modal analysis cannot be maintained in real experimental modal analysis.

**Figure 9:**
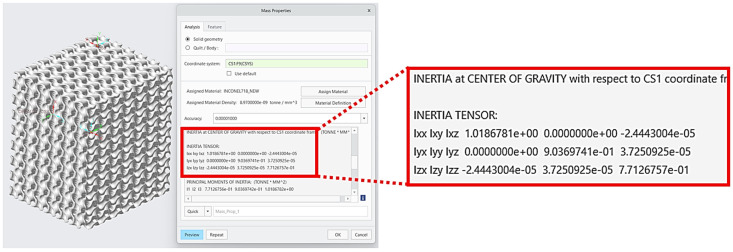
The moment of inertia specification.

As it was determined based on modal analysis that the first natural mode is the first torsional mode, that value could be calculated according to Equation (5) employing PTC Creo software. The mass moment of inertia to the *Z*-axis was specified as it is shown in Figure 9, i.e., *J* = 0.77126757 tmm^2^ (*J* = 771.26757 kgmm^2^).

Within the system with one degree of freedom and considering the lowest frequency, the stiffness of the sample could be computed according to Equation (6). Taking into account porosity as an advantage, the stiffness and damping properties can be consequently also increased using e.g., a liquid or other independent material to fill the cavities [46].

## 5. Conclusions

When designing production machines that work in specific extreme conditions—for example, at high temperatures in incinerators or industrial workshops for metals production and processing—it is imperative to ensure that such components are meticulously selected, and their quality is verified before their implementation in real operation. The mechanical properties and material selection criteria are exceedingly stringent when designing these machines. Machine components must be highly rigid, possess excellent damping properties, and be capable of withstanding high loads, which are frequently applied at high speeds and temperatures for an extended period. The load-bearing components of these devices can be equipped with cellular structures that confer extraordinary properties. Modal analysis can be employed to evaluate the quality, integrity, and reliability of components during machine operation. The stiffness of the load-bearing components can be calculated by identifying irregularities based on the specification of the natural frequencies, and the system can be prevented from resonating.

When creating, stimulating, analyzing, displaying, and evaluating the results of complex multidisciplinary systems, computer support with algorithmized procedures is used; however, it is crucial to have the skills and experience to correctly interpret the obtained results and then carefully change the system parameters to achieve the required dynamic properties of the mechanical and control systems of already existing, but primarily new products.

- In this paper, numerical and experimental modal analyses of porous specimens made of Inconel 718 alloy are presented to define the stiffness of load-bearing components. The authors pointed out the possibilities of implementing triply periodic minimal surfaces porous structures together with the specification of natural frequencies and modes of lightweight load-bearing components of machines, which can be considered a novelty.
- The analysis specified the first four natural frequencies ranging from 10 to 16 kHz. The results revealed that the first natural frequency corresponds to the first torsional frequency about the *Z* axis, the second to the first flexural mode in the XZ plane, the third to the first bending mode in the YZ plane, and the fourth to the first torsional mode about the *X* axis.
- The maximum relative error of 6.53% between both approaches in the range of quantified natural frequencies has been identified and can be considered acceptable for the given conditions. The differences that arose during the experimental modal analysis were caused by errors in the geometry of the realistic production effects and probably by careless removal from the platform. Smaller deviations in the results can also be expected due to the sensor gluing procedure.
- Although modal analysis of solid components has been used in many studies, the investigation of modal properties of the complex TPMS structures is still rare since the preparation of geometry and the FE model mesh has proved challenging.
- Taking into account porosity as an advantage, the stiffness and damping properties of the porous model with the gyroid structure can be consequently increased using, e.g., a liquid or other independent material to fill the cavities.

## Figures and Tables

**Table 1:** Material properties and ambient conditions used for FEA.

Description	Value
Young’s modulus (MPa)	1.65 × 10^5^
Poisson’s ratio	0.3
Bulk modulus (MPa)	1.375 × 10^5^
Shear modulus (MPa)	63,462
Density (kg/m^3^)	8220
Ambient temperature (°C)	21

**Table 2:** Natural frequencies obtained numerically and experimentally.

Natural Frequency	Type	FEA (Hz)	Experiment (Hz)	Relative Error (%)
No. 1	1st torsional about *Z* axis	11,078	11,801	6.53
No. 2	1st bending in XZ plane	13,523	14,193	4.95
No. 3	1st bending in YZ plane	14,562	15,138	3.96
No. 4	1st torsional about *X* axis	15,151	15,979	5.46

## Data Availability

Data are contained within the article.
